# Geometric Features-Based Parking Slot Detection

**DOI:** 10.3390/s18092821

**Published:** 2018-08-27

**Authors:** Qian Li, Chunyu Lin, Yao Zhao

**Affiliations:** Beijing Key Laboratory of Advanced Information Science and Network, Institute of Information Science, Beijing Jiaotong University, Beijing 100044, China; 17125158@bjtu.edu.cn (Q.L.); yzhao@bjtu.edu.cn (Y.Z.)

**Keywords:** around view, geometric features, parking slot detection

## Abstract

In this paper, we propose a parking slot markings detection method based on the geometric features of parking slots. The proposed system mainly consists of two steps, namely, separating line detection and parking slot entrance detection. First, in the separating line detection stage, we propose a line-clustering method based on the line segment detection (LSD) algorithm. Our detecting and line-clustering algorithm can detect the separating lines that contain a pair of parallel lines with a fixed distance in a bird’s eye view (BEV) image under diverse lighting and ground conditions. Consequently, parking slot candidates are generated by pairing the separating lines according to the width of the parking slots. In the parking slot entrance detection process, we propose a multiview fusion-based learning approach that can increase the number of training samples by performing a perspective transformation on the acquired BEV images. The proposed method was evaluated using 353 BEV images covering diverse parking slot markings. Experiments show that the proposed method can recognize typical perpendicular and parallel rectangular parking slots, and a precision of 97.4% and recall of 96.6% are achieved.

## 1. Introduction

In recent times, interest in automotive technology and the popularity of automobiles have been increasing. In this context, parking in a narrow space is a difficult task for many beginners. As a result, intelligent transport systems have emerged. However, there are still many problems that need to be solved in this research field. An automatic parking system [1,2,3] consists of three parts: target position designation, path planning and path tracking. Target position designation is the most important component of intelligent transport. Various methods of identifying the target position designation have been proposed in the literature and they can be classified into four categories: free space-based [4,5,6,7,8,9,10,11], parking slot marking-based [12,13,14,15,16,17,18,19,20,21,22,23,24,25], interface-based [26,27,28] and infrastructure-based.

Of these, the free space-based method is extensively researched but has a drawback; if there are no adjacent vehicles, the method cannot work.

Compared with the other approaches, the parking slot marking-based method is able to recognize parking slots more accurately because its recognition process does not depend on the presence of adjacent vehicles, but on parking slot markings. Furthermore, it does not need assisted sensors, such as short-range radar and scanning laser radar. The previous approaches can roughly be generalized as follows. The method proposed in [12] can recognize various types of parking slot markings in a fully automatic manner. First, this method simultaneously finds parallel line pairs whose gradient orientations are opposite when detecting the separating lines. Next, the method utilizes the line detector and Harris corner detector to recognize the entrance positions. However, traditional vision-based features are sensitive to noise when the lines or corners are faint and distorted in the Around View Monitor (AVM) images. The methods in [13,14] are not robust. They cannot give the desired result under complex light intensity and ground conditions. The method in [15] also finds parallel lines with a fixed distance using line segment detection (LSD) based on edge image, but it has a drawback that the straight line will be divided into many small segments, leading to an increase in false detection. The method in [16] is a data-driven learning-based approach called PSD_L_, which detects the marking-points first and then infers the valid separating marking lines using six Gaussian line templates. If the marking-point is wrongly detected, the parking slot will not be found correctly, and the detection accuracy will decrease.

By contrast, offline detection models always provide very accurate results. However, the learning-based approach is generally very time consuming because it trains multiple detectors and the marking point can be in any direction. We will review the literature in more detail in the next section.

Therefore, in order to address the aforementioned concerns, this paper proposes a geometric features-based method that can achieve much better results even if the parking slot markings are presented in various lights (dim, strong) and ground conditions (bricked, curved, blurred, marked) in a fully automatic manner. The proposed method first detects the separating lines by utilizing a line-clustering approach based on the previous LSD algorithm. Parking slot candidates are formed by pairing the separating lines according to geometric features of the parking slot. Finally, this paper detects the entrance using a line-based and learning-based method. If the number of guide lines is higher than 1 by using the line-based method, we use the learning-based method to redetect the guide line. The method was evaluated on a database containing various complex conditions, and it achieves a 96.6% recall and 97.4% precision.

The contributions of this paper are as follows:

(1) It suggests a line-clustering method, which, compared with previous line detection methods including the distance transform and the Hough line detector, has more robust detection under varied illumination conditions.
(2) As a large-scale dataset is still lacking, we propose a multiview fusion method to increase the training set.
(3) Previous learning-based methods need to train multiple marking-point detectors, whereas in this paper, we only train one detector and identify the shape of a parking marking-point by using a convex defect algorithm, which increases the number of the true positives and gives more robust detection results.

The rest of this paper is organized as follows. Section 2 explains the related research. Section 3 describes the proposed scheme. Section 4 presents experimental results. Finally, this paper is concluded with a summary in Section 5.

## 2. Related Research

Given that this paper is concerned with a vision-based approach, the literature review in this section focuses on slot-marking-based methods. All approaches using this method utilize cameras that can detect the parking slot markings painted on the ground. These methods are categorized into two streams, the semiautomatic ones and automatic ones.

In 2000, Xu et al. [17] utilized the color of parking slot’s markings to identify the pixels of these markings from images. In 2006, Jung et al. [18] recognized marking line-segments using directional intensity-gradient on a line lying from seed-point to camera. This method is semi-automatic and requires manual selection of the parking slots. Our method is fully automatic and there is no need to manually select the parking space. In 2011, Schmid et al. [19] proposed the use of a hierarchical three-dimensional occupancy grid for the detection of parking spaces. The presented approach derives the distance to obstacles and walls and is thus able to represent the free spaces that form parking spaces. In 2012, Suhr et al. [20] proposed a novel fully automatic method for recognizing various slot markings in image sequences acquired by an Around View Monitor (AVM) system. In 2013, Suhr et al. [21] recognized various types of parking slot markings by modeling them as a hierarchical tree structure in a fully automatic method. In 2014, Wang et al. [14] proposed an automatic parking method through a bird’s eye view vision system, which extracts the features of the parking spaces using a Radon transform-based method, after which double circular trajectory planning and a preview control strategy are utilized to realize autonomous parking. Suhr et al. [22] proposed a vacant parking slot detection and tracking system that fuses the sensors of an Around View Monitor (AVM) system and an ultrasonic sensor-based automatic parking system. In 2016, Lee et al. [23] proposed a robust parking slot detection algorithm based on the line-segment-level clustering method. The proposed algorithm consists of line-segment detection using the proposed directional density-based spatial clustering algorithm (Directional-DBSCAN) and slot detection using slot pattern recognition. The Directional-DBSCAN algorithm robustly extracts lines even when they are short and faint. Lee et al. [24] presented a camera-based available parking-slot recognition algorithm based on slot-context analysis. This method can handle diverse available parking-slot conditions. The method proposed a new methodology of extracting and associating line-markings to recognize the parking slot, and via the histogram of gradient and frequency-magnitude features, the slot-occupancy could be classified. Suhr et al. [25] proposed a parallel line-based method that utilizes random sample consensus(RANSAC) and distance transform (DT)-based chamfer matching to detect parking slot markings.

The proposed method is a fully automatic approach. It can reliably recognize parking slot markings under diverse lighting and ground conditions. The proposed multiview fusion-based learning approach can also increase the number of training samples and decrease the false detection results.

## 3. Proposed Scheme

**Figure 1:**
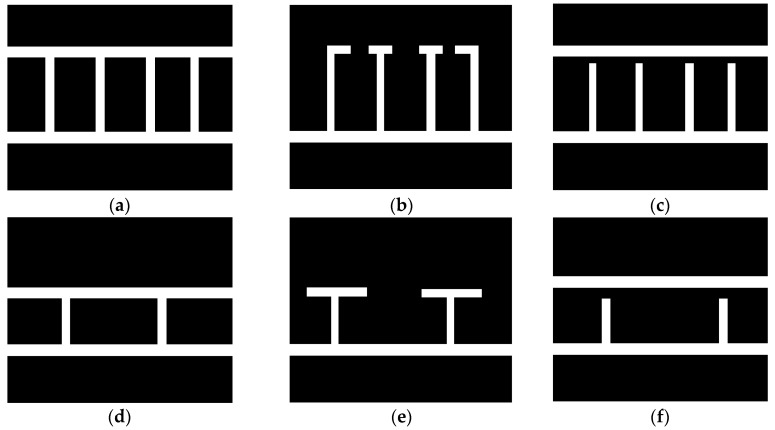
Typical types of parking slot markings. (**a**–**c**) are the perpendicular parking slots; (**d**–**f**) are the parallel parking slots.

The proposed method utilizes a geometric features-based approach. As shown in Figure 1, parking slot markings consist of one guide line and several parallel separating lines with a fixed distance. Each parking slot is distinguished by two separating lines perpendicular to the guide line. In addition, there are two kinds of guide lines: a continuous straight line and T-shaped or L-shaped marking points. According to the inherent geometric constraints of the parking slots, the proposed method detects the separating lines using line clustering [29,30,31,32], which can remove the false lines, and then detects the guide line using a learning-based method. The first row of Figure 1 shows perpendicular parking slots. The second row of Figure 1 shows parallel parking slots corresponding to images with the same guide line as the first row of Figure 1. Compared with the previous approach, the proposed method is more robust under complex lighting conditions.

**Figure 2:**
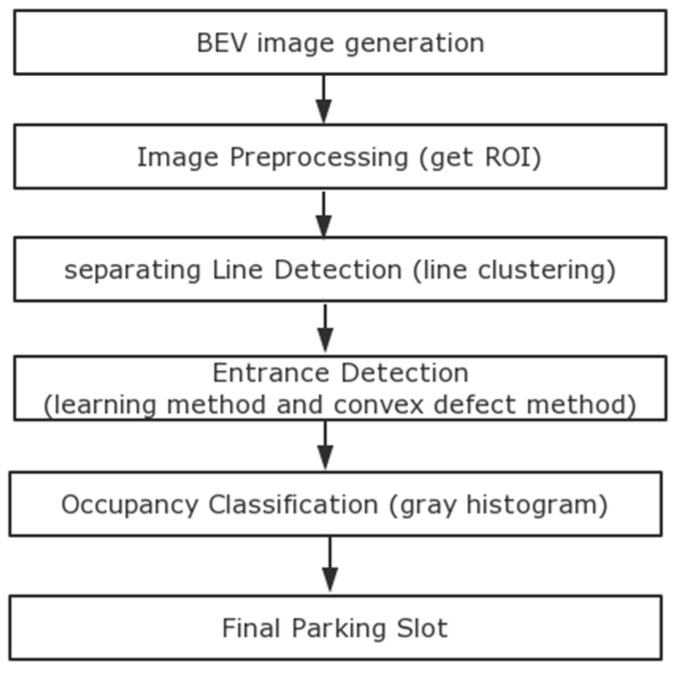
Overall block diagram of the proposed scheme.

**Figure 3:**
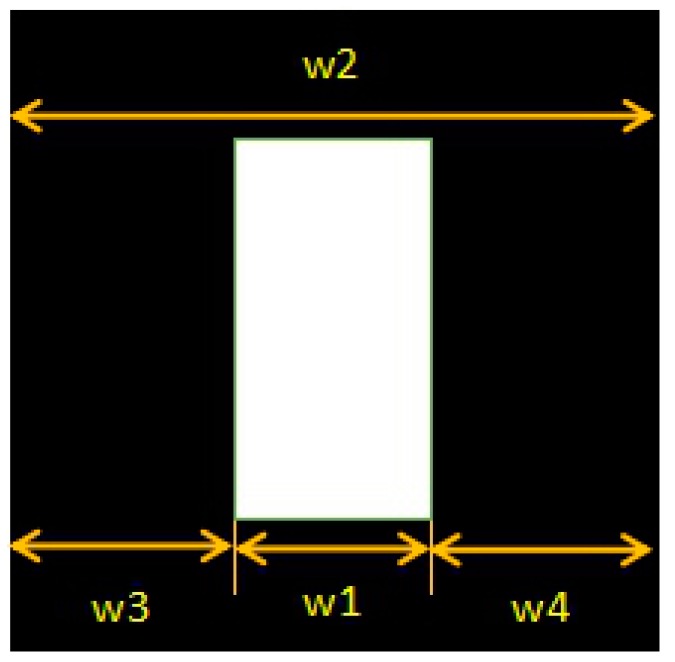
Definition method of the region of interest (ROI).

Figure 2 illustrates an overall block diagram of the proposed scheme. For a given bird’s eye view (BEV) image, we perform preprocessing first. Under normal circumstances, when the driver is driving in the parking lot, parking slot markings will appear on the left and right sides of the driver. Therefore, we proposed a method for obtaining the region of interest (ROI) instead of detecting parking slot markings on the entire image, which can reduce our overall process time. Figure 3 illustrates the method of defining the ROI. w_1_ indicates the width of the car in the AVM image, w_2_ indicates the width of the AVM image, w_3_ and w_4_ are the widths of the left ROI and right ROI, respectively. The geometric constraint is as follows: w_3_ = w_4_ = (w_2_ − w_1_)/2. The main steps of the proposed method are as follows: the first part is to detect parking separating lines, which have a pair of parallel lines with a fixed distance in a surround view image. Next is to detect the parking slot entrance. The process utilizes a multiview fusion-based learning approach rather than the previously used Harris corner detector method in [12]. For the occupancy classification, we use the method proposed in [15].

**Figure 4:**
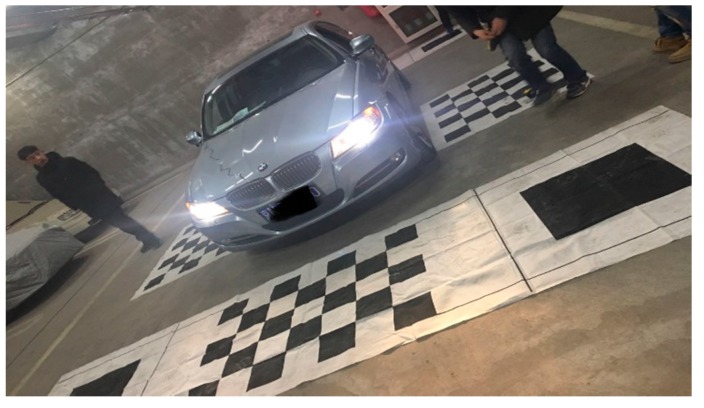
Camera calibration of the Around View Monitor (AVM) system.

Our method is based on an AVM [33] system. The AVM system consists of four fisheye cameras equipped at four positions, that is, front, rear, left and right side of the car. A view transform and image mosaicking are performed on the system, which provide a so-called ‘bird’s eye view’ image at the top of the vehicle. As the AVM system is very mature, it is not described in detail here. The system is shown in Figure 4.

### 3.1. Image Preprocessing

**Figure 5:**
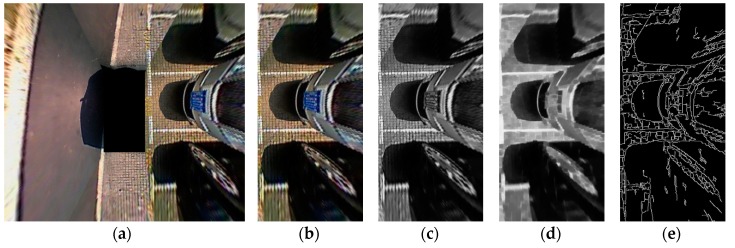
Preprocessing results. (**a**) Original AVM image; (**b**) ROI image; (**c**) Gray image; (**d**) Morphological filtering result; (**e**) Edge image.

The quality of the image directly affects the accuracy of the recognition algorithm. Therefore, before parking slot detection, preprocessing is required. The purpose of image preprocessing is to eliminate irrelevant information in images, recover useful information and enhance the detectability of related information. In this study, the preprocessing is conducted as follows: First, the proposed method obtains the ROI, we do not need to focus on non-ROIs, and the method reduces overall processing time. In addition, the colorful images are converted to their grayscale versions. At last, in order to detect the parking slot markings more accurately, it is necessary to perform morphological filtering and image edge detection, as morphological filtering can produce smoother edges. In this study, the edge image is generated by the Canny detector. Figure 5 shows the preprocessing results.

### 3.2. Separating Line Detection

In this section, we first use the LSD approach to detect the separating lines as it is faster than the Hough transform method. The LSD algorithm is mainly divided into three parts: extraction of the line-support region, rectangular approximation and line segment validation. However, LSD is an algorithm for local extraction of lines, and there are some disadvantages of the local algorithm: two intersecting lines will be split into four segments at the intersection, and because of the self-growth characteristics of the local detection algorithm, it is often split into multiple straight lines for reasons such as occlusion of long line segments and local blurring. To resolve the problems mentioned above, we propose a line-clustering method based on the detection result of LSD, which can accurately recognize the parking slot markings under diverse lighting and ground conditions.

**Figure 6:**
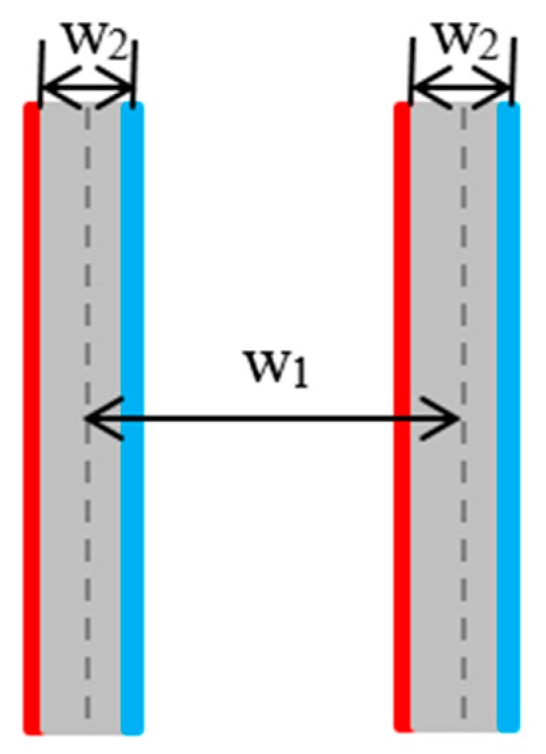
Geometric features of a parking slot.

**Figure 7:**
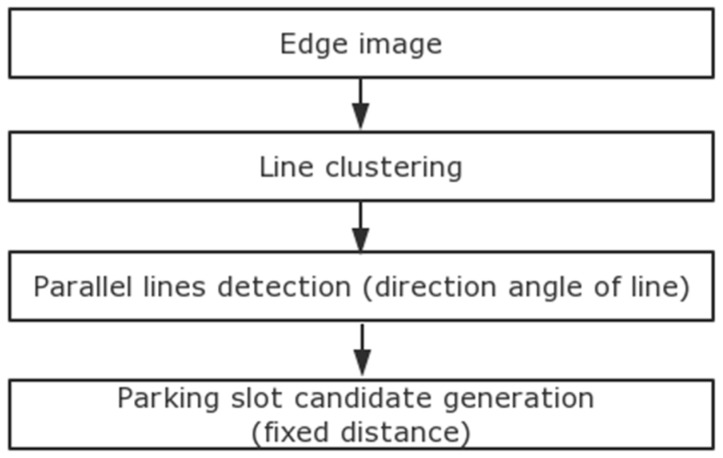
Block diagram of separating line detection.

There are two main geometric features of a parking slot, as shown in Figure 6. One is that a separating line is composed of two parallel lines with fixed distance w_2_. If the direction angles of the red line and blue line are the same and the distance between the red line and blue line is within the range of w_2_, the separating line will be detected. The other is that two separating lines can form a parking slot if their distance is within a certain range of w_1_. Different from the method in [12], the proposed method ignores the property that the gradient of two parallel lines are opposite. The process utilizes a line-clustering method based on previous preprocessing results, and it contains four main steps. Figure 7 shows the block diagram of this process.

**Algorithm 1:** Rules of line fittingInput: {L}, ε, θ Output: { lineclasses } 1.foreach line $L_{i}$ in {L} do 2. initialize lineclass; 3. flag = false; 4. foreach lineclass in { lineclasses } 5.      if distance < ε, angle < θ 6.           flag = true; 7.              calculate new start point and end point of new lineclass 8.           break; 9.      end 10.     if flag = false 11.        pushback lineclass into {lineclasses}; 12.      end 13.end 14.end

**Figure 8:**
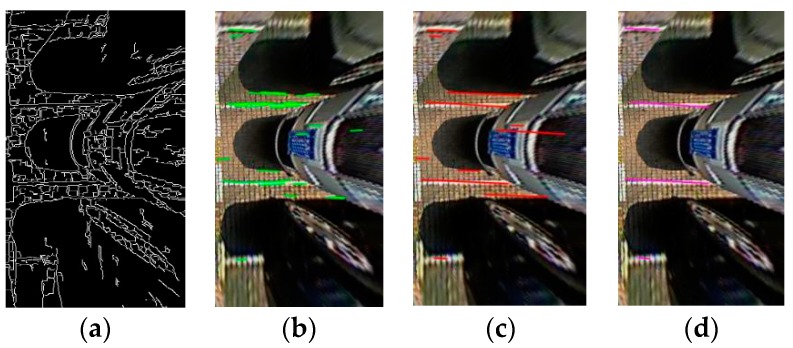
Separating line detection results. (**a**) Edge image; (**b**) Detection by line segment detection (LSD); (**c**) Line-fitting result; (**d**) Parking slot candidates.

For a given edge image, as shown in Figure 8a, the LSD algorithm is performed on it first. According to the detection result of LSD, we set a length threshold value. If the length of the straight-line segment is less than the given threshold, the straight line is not retained; thus, the efficiency can be improved in later processing. Figure 8b shows the detection result by the LSD method. The next step is line clustering. The existing clustering method is mainly used to solve the matching and pattern recognition of complex trajectories. The method in [29] addresses the problem of coalition formation among Machine-to-Machine communication type devices and the resource management. Methods in [30,31] are applied to the radio network architecture. In [32], a simple line clustering method was presented, it adopts entropy theory and the probability distribution function for parameter selection to acquire clustering results. In this paper, we proposed a line clustering method, and the line-clustering rules are presented in Algorithm 1. The average running time of Algorithm 1 is 0.4 s. Here, {L} denotes all lines detected by the LSD approach, ε and θ are the distance and angle threshold, respectively. According to the results of the line clustering shown in Figure 8c, we calculate the direction angle of each straight line. In practice, for the sake of experimental validity, if the direction angle is between −8 and 8 degrees, we regard the straight lines as parallel lines. To the parallel lines obtained above, if the distance satisfies the width of a parking slot, it can be regarded as a parking slot candidate, as shown in Figure 8d. Through the geometrical constraints, we can remove false separating lines. Compared with the previous LSD method in [15], the line-clustering approach gains more accurate and robust detection results.

### 3.3. Parking Slot Entrance Detection

**Figure 9:**
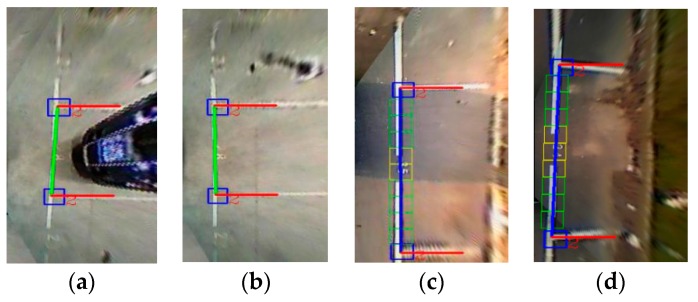
Entrance detection results. (**a**,**b**) are the entrance detection results of perpendicular parking slots; (**c**,**d**) are the entrance detection results of parallel parking slots.

As parking slot candidates do not contain parking direction information, they cannot help the driver to park the car conveniently and safely. This study utilizes a line-based and machine learning-based approach that deals with typical rectangular parking slots. According to the geometric characteristics of the parking slots, we first utilize the line clustering-based algorithm to detect the guide line that is perpendicular to the separation lines. If the number of detected guide lines is higher than 1, we use the learning-based method to redetect the T-shaped or L-shaped marking-points at the guide line. Through the positions of the marking-points, we can obtain the true guide line and remove the false guide lines, as shown in Figure 9.

**Figure 10:**
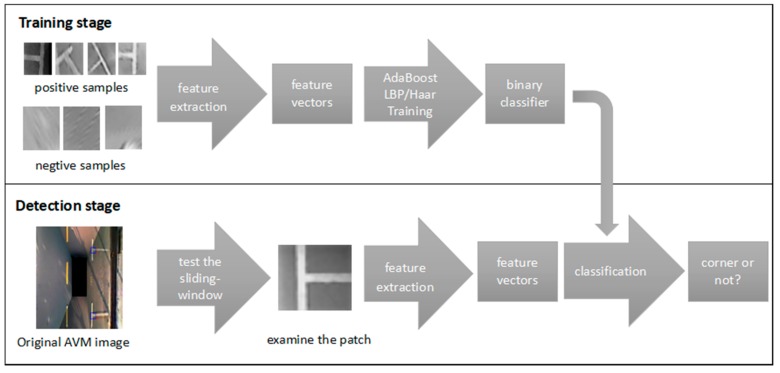
Training and detection stages of the proposed process.

A typical learning-based method of the marking-point recognition system is shown in Figure 10. The system takes the T-shaped or L-shaped marking-point as the input and uses the classification result as the output. The marking-point detection of a parking slot is to locate and segment the marking-point from a complex background. Generally, it needs to be preprocessed, and this kind of preprocessing has considerable influence on the recognition performance. The preprocessing mainly needs to normalize the size of the square area obtained and convert the color image to a grayscale image to speed up the processing. Feature extraction and classification recognition are the core of the entire system and directly determine the performance of the final identification. In general, feature extraction is actually a process of linear or nonlinear transformation coding of the image patch. It causes the image patch to be represented by lower-dimensional data to reduce the calculation cost and causes dimensionality reduction. Classification recognition is used to select one or more classifier algorithms for target classification or identification. The proposed method of machine learning is a pattern recognition technology, and it is used to classify the data.

Therefore, before designing the classifier, we must first prepare a large number of comprehensive training samples and test samples that can cover various situations. Then, the training samples are analyzed using statistical methods, which mainly involves analyzing the characteristics of the samples and the distribution of sample feature values and finally classifying the samples correctly.

The training samples are divided into positive samples and negative samples. A positive sample is an image that contains the marking-point to be measured and it can be generated from a picture that contains the object to be measured, or it can be created from a series of marked figures. A negative sample is any image that does not include the marking-point to be measured. Negative samples must be manually prepared. The test samples are used to check the classifier’s rationality problem. They need to modify the classifier according to the result of the test samples. This is an iterative process. However, because of the lack of sample sets in the field, and previous studies being based on AVM images, they could only handle one kind of view. Therefore, we propose a multiview-fusion method in which the backward perspective transformation is performed on the bird’s eye view images, which considerably increases the number of training samples. The proposed method has higher applicability than those in previous studies.

Considering the real-time, fault tolerance and scalability requirements, we utilize the cascade classifier, Haar feature [34] and LBP feature [35]. The AdaBoost [36] method trains the same classifier (weak classifier) for different training sets and then combines these classifiers obtained on different training sets to form a stronger final classifier (strong classifier).

**Figure 11:**
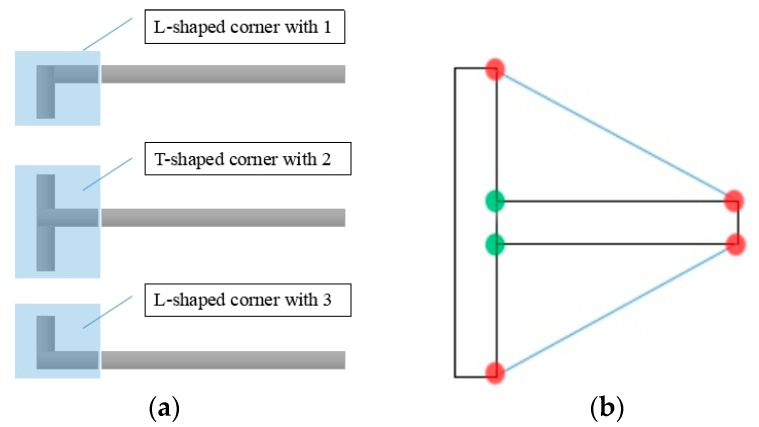
(**a**) Typical marking-points; (**b**) Convex defect method.

**Figure 12:**
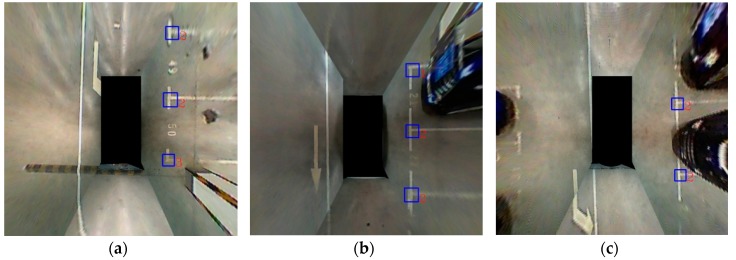
Detection results of the learning method. (**a**–**c**) are the detection results of perpendicular parking slots.

Compared with earlier methods proposed in [12,16], we only train one classifier, that is, all the T-shaped and L-shaped corners are considered positive samples, and finally the shape of the marking-point is judged by using the convex defect method, which is illustrated in Figure 11. In Figure 11b, the black line indicates the detected contour, the blue line indicates the convex hull, the red dots indicate the start point and the end point, the green dots indicate the farthest points, and the area between the blue line and the two black vertical lines is called a defect. The judgment rules are presented in Algorithm 2, and the average running time of Algorithm 2 is 0.9 s. Here, i denotes the number of defects; defects (i) (0) and defects (i) (1) indicate the start point and the end point respectively; defects (i) (2) indicates the farthest point; and defects (i) (3) represents the distance between the farthest point to the convex hull. Distance is a predefined threshold. The approach presented in this paper has a more robust detection result. In Figure 12, the detection results are shown.

**Algorithm 2:** Rules of convex defect1.foreach defects (i) in defects 2.   if defects (i) (3) > distance 3.        then compute angle of defects 4.              if angle>=75 &&angle<=120 5.                   then push the defects (i) into the finalDefects. 6.              end 7.   end 8.end 9.if the size of finalDefects is 1 10.        then p1=farthest point, p2=average of start and end points 11.        if p2.y<p1.y 12.             return 3 13.        end 14.        else if p2.y>p1.y 15.           return 1 16.        end 17.        else 18.        return false 19.        end 20.end 21.else if the size of defects is 2 22.      return 2 23.end 24.end 

These results in Figure 12 show that the machine learning-based method works well. It can correctly recognize three kinds of marking points even in a variety of lighting conditions. The blue box indicates the position of the marking point, and the number next to it indicates the type of the corner. Thus, 1 and 3 indicate the L-shape, and 2 indicates the T-shape.

### 3.4. Parking Slot Occupancy Classification

**Figure 13:**
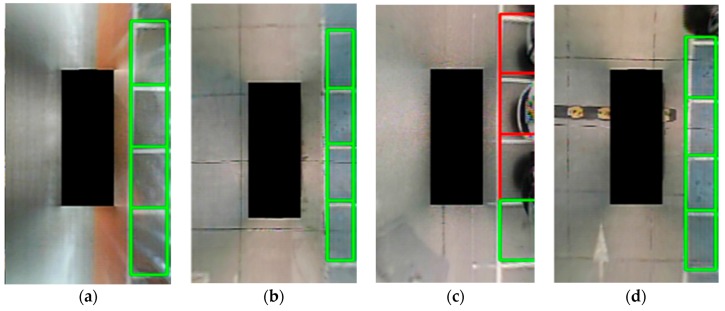
Occupancy classification result. In (**a**–**d**), the green boxes indicate that the parking slots are empty, while the red boxes indicate that the parking slots are occupied.

This is the last step of the method proposed by the dissertation. At present, most parking slot detection algorithms based on traditional vision have not classified the occupancy of parking spaces. In fact, the main purpose of the parking system is to find an effective empty parking slot. The method in [12] classified the occupancy based on the ultrasonic sensors. Unlike [12], the method presented in [15] adopted the gray histogram to classify the parking slots occupancy. In this study, we do not propose a new classifying method; we utilize the gray histogram. The process is not our contribution. Figure 13 shows the occupancy classification result. A green box indicates that the parking space is empty, while a red box indicates that the parking space is occupied.

## 4. Experimental Results

### 4.1. Description of the Database

To evaluate the method proposed in this paper, we conducted experiments using the test database presented in [16]. There are 353 images included in this database, which consist of 258 outdoor parking slots and 95 underground parking slots. Images in this test database were captured by fisheye camera, and their resolution is 600 × 600 pixels. This test database covers a variety of complex lighting and ground conditions, such as dim light in basement scenes, shadow in outdoor parking lots and large numbers of stains on the road surface.

**Figure 14:**
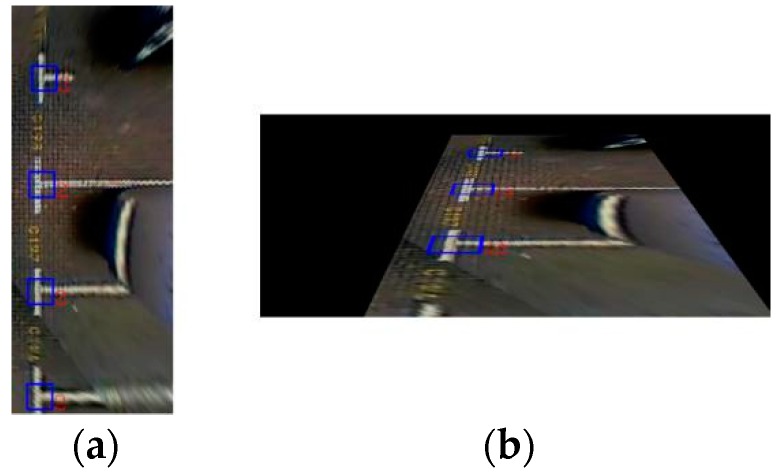
Result of a perspective transformation. (**a**) bird’s eye view (BEV) image; (**b**) Perspective transformation image.

**Figure 15:**
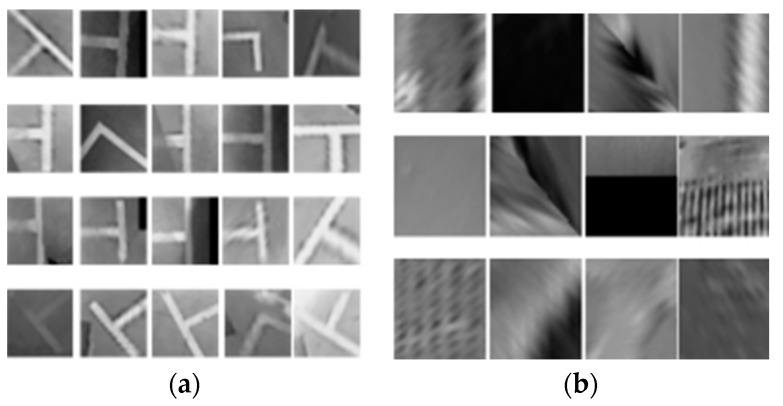
Training dataset. (**a**) Positive samples; (**b**) Negative samples.

In terms of the training database, we marked 5100 images. The images in the training database were also captured by an AVM system, and the resolution is the same as the test database. To increase the number of the training samples, we performed a perspective transformation on the acquired AVM images. Figure 14a shows the example image of the original BEV image. Figure 14b shows the perspective transformation image corresponding to the BEV image. We obtained a total of 16,690 positive samples and 30,000 negative samples. The size of the training samples is 30 × 30 and 40 × 40. The negative samples are obtained by randomly cropping an image patch containing non-parking slots in the marked pictures. All samples are converted to grayscale, as shown in Figure 15.

### 4.2. Performance Evaluation

To compare with the previous learning-based method [16], we make use of two metrics: recall and precision, as shown in Equation (1).

(1) $$\mathrm{precision}=\frac{\mathrm{true}\;\mathrm{positives}}{\mathrm{true}\;\mathrm{positives}+\mathrm{false}\;\mathrm{positives}}\;\mathrm{recall}=\frac{\mathrm{true}\;\mathrm{positives}}{\mathrm{true}\;\mathrm{positives}+\mathrm{false}\;\mathrm{negtives}}$$

**Figure 16:**
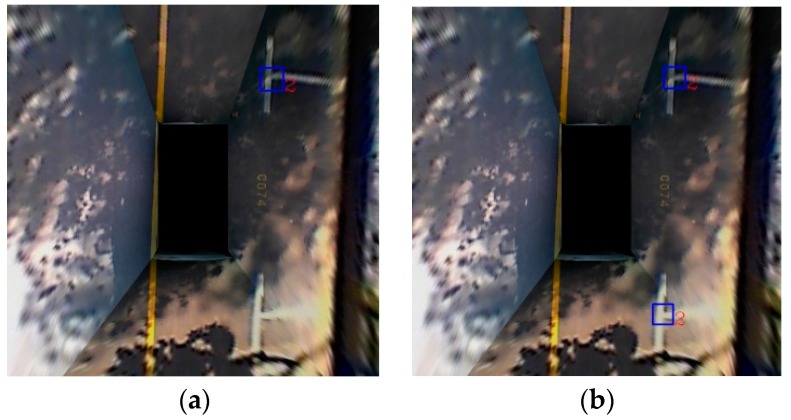
Comparison result between the BEV classifier and the MV classifier. (**a**) is the detection result using the BEV classifier; (**b**) is the detection result using the MV classifier.

To verify that the proposed multiview-fusion method is more efficient, we trained two classifiers. For the training sample of one classifier, we chose to use only the bird’s-eye view perspective. For the training sample of another classifier, we used the multiview fusion method. We defined the classifier trained from bird’s-eye view as the BEV classifier, and the classifier trained from multiview fusion as the MV classifier. Figure 16 shows the comparison result between the BEV classifier and the MV classifier. As seen in Figure 16, the detection result using the MV classifier is better than that of the BEV classifier.

Table 1 lists the comparison results of learning-based marking-point detection between the MV classifier and the BEV classifier using outdoor images in the test database. In this table, #marking point, #TP and #FP indicate the number of existing marking points, correctly recognized marking points, and incorrectly recognized marking points, respectively. The MV classifier gives a recall of 96.8% and a precision of 98.2%, while the BEV classifier gives a recall of 94.0% and a precision of 96.0%. This reveals that the MV classifier outperforms the BEV classifier by 2.2% and 2.8% in terms of precision and recall, respectively. This is because, through perspective transformation, the number of training samples has doubled, which made the performance of the classifier more reliable.

**Figure 17:**
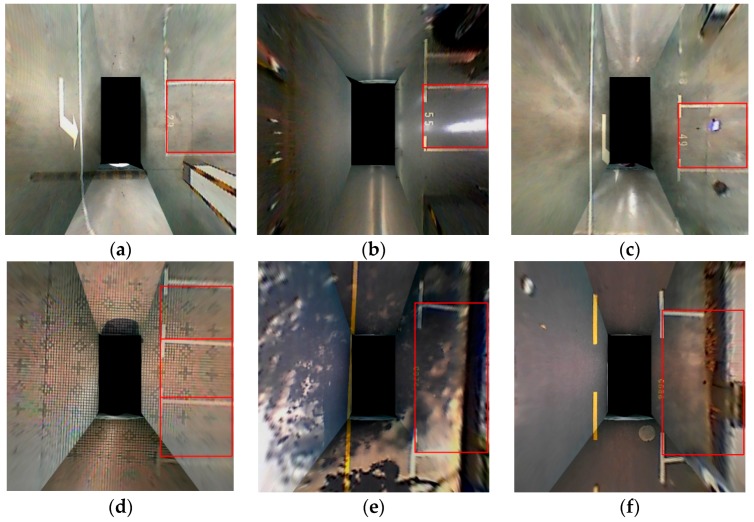
(**a**–**c**) are the detection results using the underground test database; (**d**–**f**) are the detection results using the outdoor test database.

Table 2 presents the comparison results between the previous method in [16] and the proposed method, using the underground test database. In this table, #slots, #TP and #FP indicate the number of existing slots, correctly recognized slots and falsely recognized slots, respectively. The proposed method gives 96.8% for recall and precision, while the method in [16] gives a recall of 93.7% and a precision of 96.7%. This reveals that the proposed method outperforms the previous method by 0.1% and 3.1% in terms of precision and recall, respectively. This is mainly because, in the underground parking environment, the lighting is dim and there is strong reflected light on the ground. The proposed method can better detect the parking line under these lighting conditions. Table 3 shows the comparison results between the previous method in [16] and the proposed method, using the outdoor test database. The proposed method gives a 1.1% lower precision but 3.1% higher recall than the previous method. This is because the proposed algorithm is able to detect more true positives. Table 4 presents the comparison results between the previous method and the proposed method using the combined test database. On average, the proposed method achieves 97.5% for both recall and precision while the method in [16] gives a precision of 98.2% and a recall of 94.3%. In terms of the precision, the proposed method gives just 0.7% lower precision than the previous method described in [16]. This is because the previous method produces no parking slot when it cannot detect the marking point, which means that the number of false positives is lower than in the proposed method. In this case, we can compare the recall rates. The best method should achieve the highest recall rates. Based on Table 4, it can be seen that the proposed method gives a higher recall by 3.2% compared to the method in [16], which reveals that the proposed method outperforms the previous method. Figure 17 gives the final parking slot detection results under a variety of lighting conditions. These results show that the proposed method works well. It detects separating lines using a line-clustering method according to the geometric constraints, and it detects the entrances using multiview fusion-based learning method, which can increase the number of true positives.

## 5. Conclusions

Parking slot detection is an important aspect of an automatic parking system, but an accurate vision-based parking slot detection method has yet to be developed. In this paper, we proposed a geometric features-based parking-slot-marking detection approach, which involves three notable aspects. First, we proposed a line clustering method that can recognize the parking slot markings under diverse complex lighting and ground conditions. Second, we proposed a multiview fusion-based method, which can increase the number of training samples and improve the accuracy of the detection result. The experiment was evaluated on our test database, and the method outperforms the single bird’s eye view method. Finally, we only trained one detector, and the convex defect was utilized to identify the T-shape or L-shape of the marking points. Since the running time of our method cannot meet the real-time requirement, we will try to reduce the complexity of our scheme later. In addition, we intend to use deep learning in parking slot detection, because deep learning has high accurate detection performance. However, deep learning requires large number of labeled samples that can be accumulated in the future.

## Figures and Tables

**Table 1:** Performance comparison of two marking-point detection methods.

Type	#Marking Point	#TP	#FP	Precision	Recall
BEV classifier	284	267	11	96.0%	94.0%
MV classifier	284	275	5	98.2%	96.8%

**Table 2:** Performance comparison of the two methods using the underground database.

Method	#Slot	#TP	#FP	Precision	Recall
Method in [16]	95	89	3	96.7%	93.7%
Proposed method	95	92	3	96.8%	96.8%

**Table 3:** Performance comparison of the two methods using the outdoor database.

Method	#Slot	#TP	#FP	Precision	Recall
Method in [16]	258	244	3	98.8%	94.6%
Proposed method	258	252	6	97.7%	97.7%

**Table 4:** Performance comparison of the two methods using the combined database.

Method	#Slot	#TP	#FP	Precision	Recall
Method in [16]	353	333	6	98.2%	94.3%
Proposed method	353	344	9	97.5%	97.5%

## References

[1] Rahayu Y., Mustapa F.N. (2013). A secure parking reservation system using GSM technology. Int. J. Comput. Commun. Eng..

[2] Mainetti L., Palano L., Patrono L., Stefanizzi M.L., Vergallo R. Integration of RFID and WSN technologies in a smart parking system. Proceedings of the 22nd International Conference on Software, Telecommunications and Computer Networks (SoftCOM).

[3] Gupta A., Kulkarni S., Jathar V., Sharma V., Jain N. (2017). Smart Car Parking Management System Using IoT. Am. J. Sci. Eng. Technol..

[4] Ban X., Chu L., Herring R., Margulici J.D. (2011). Sequential modeling framework for optimal sensor placement for multiple intelligent transportation system applications. J. Trans. Eng..

[5] Kim W., Kim D., Yi K., Kim H.J. (2012). Development of a path-tracking control system based on model predictive control using infrastructure sensors. Veh. Syst. Dyn..

[6] Jeong S.H., Choi C.G., Oh J.N., Yoon P.J., Kim B.S., Kim M., Lee K.H. (2010). Low cost design of parallel parking assist system based on an ultrasonic sensor. Int. J. Autom. Technol..

[7] Alonso L., Milanes V., Torre-Ferrero C., Godoy J., Oria J.P. (2011). Ultrasonic sensors in urban traffic driving-aid systems. Sensors.

[8] Kim H., Lee J.H., Kim S.W., Ko J.I., Cho D. (2001). Ultrasonic vehicle detector for side-fire implementation and extensive results including harsh conditions. IEEE Trans. Intell. Transp. Syst..

[9] Carullo A., Parvis M. (2001). An ultrasonic sensor for distance measurement in automotive applications. IEEE Sens. J..

[10] Agarwal V., Murali N.V., Chandramouli C. (2009). A Cost-Effective Ultrasonic Sensor-Based Driver-Assistance system for Congested Traffic Conditions. IEEE Trans. Intell. Transp. Syst..

[11] Zhou J., Navarro-Serment L.E., Hebert M. Detection of parking spots using 2D range data. Proceedings of the 15th IEEE International Conference on Intelligent Transportation Systems.

[12] Suhr J.K., Jung H.G. (2018). A Universal Vacant Parking Slot Recognition System Using Sensors Mounted on Off-the-Shelf Vehicles. Sensors.

[13] Jung H.G., Kim D.S., Yoon P.J., Kim J. Parking slot markings recognition for automatic parking assist system. Proceedings of the IEEE Intelligent Vehicles Symposium.

[14] Wang C., Zhang H., Yang M., Wang X., Ye L., Guo C. (2014). Automatic Parking Based on a Bird’s Eye View Vision System. Adv. Mech. Eng..

[15] Li L., Li C., Zhang Q., Guo T., Miao Z. Automatic parking slot detection based on around view monitor (AVM) systems. Proceedings of the 9th IEEE International Conference on Wireless Communications and Signal Processing.

[16] Zhang L., Li X., Huang J., Shen Y., Wang D. (2018). Vision-Based Parking-Slot Detection: A Benchmark and a Learning-Based Approach. Symmetry.

[17] Xu J., Chen G., Xie M. Vision-guided automatic parking for smart car. Proceedings of the IEEE Intelligent Vehicles Symposium.

[18] Jung H.G., Kim D.S., Yoon P.J., Kim J. Structure analysis based parking slot marking recognition for semi-automatic parking system. Proceedings of the Joint IAPR International WorkShops on Statistical Techniques in Pattern Recognition and Structural and Syntactic Pattern Recognition.

[19] Schmid M.R., Ates S., Dickmann J., Hundelshausen F., Wuensche H.J. Parking space detection with hierarchical dynamic occupancy grids. Proceedings of the IEEE Intelligent Vehicles Symposium.

[20] Suhr J.K., Jung H.G. Fully-automatic recognition of various parking slot markings in Around View Monitor (AVM) image sequences. Proceedings of the 15th IEEE International Conference on Intelligent Transportation Systems.

[21] Suhr J.K., Jung H.G. (2013). Full-automatic recognition of various parking slot markings using a hierarchical tree structure. Opt. Eng..

[22] Suhr J.K., Jung H.G. (2014). Sensor fusion-based vacant parking slot detection and tracking. IEEE Trans. Intell. Transp. Syst..

[23] Lee S., Hyeon D., Park G., Baek I.J., Kim S.W., Seo S.W. Directional-DBSCAN: Parking-slot detection using a clustering method in around-view monitoring system. Proceedings of the 2016 IEEE Intelligent Vehicles Symposium.

[24] Lee S., Seo S.W. (2016). Available parking slot recognition based on slot context analysis. IET Intell. Transp. Syst..

[25] Suhr J.K., Jung H.G. (2016). Automatic parking space detection and tracking for underground and indoor environments. IEEE Trans. Ind. Electron..

[26] Jung H.G., Lee Y.H., Kim J. (2010). Uniform user interface for semiautomatic parking slot marking recognition. IEEE Trans. Veh. Technol..

[27] Jung H.G., Kim D.S., Yoon P.J., Kim J. Two-touch type parking slot marking recognition for target parking position designation. Proceedings of the 2008 IEEE Intelligent Vehicles Symposium.

[28] Jung H.G. (2013). Semi-automatic parking slot marking recognition for intelligent parking assist systems. J. Eng..

[29] Tsiropoulou E.E., Paruchuri S.T., Baras J.S. Interest, energy and physical-aware coalition formation and resource allocation in smart IoT applications. Proceedings of the 51st Annual Conference on Information Sciences and Systems (CISS).

[30] Gerla M., Tsai T.C. (1995). Multicluster, mobile, multimedia radio network. Wirel. Netw..

[31] Lin C.R., Gerla M. (1997). Adaptive clustering for mobile wireless networks. IEEE J. Sel. Area Commun..

[32] He B., Zhang Y., Chen Y., Gu Z. (2018). A Simple Line Clustering Method for Spatial Analysis with Origin-Destination Data and Its Application to Bike-Sharing Movement Data. ISPRS Int. J. Geo-Inf..

[33] Gao Y., Lin C., Zhao Y., Wang X., Wei S., Huang Q. (2018). 3-D Surround View for Advanced Driver Assistance Systems. IEEE Trans. Intell. Transp. Syst..

[34] Saeed A., Al-Hamadi A., Ghoneim A. (2015). Head Pose Estimation on Top of Haar-Like Face Detection: A Study Using the Kinect Sensor. Sensors.

[35] Nguyen D.T., Pham T.D., Baek N.R., Park K.R. (2018). Combining Deep and Handcrafted Image Features for Presentation Attack Detection in Face Recognition Systems Using Visible-Light Camera Sensors. Sensors.

[36] Chen Y., Dou P., Yang X. (2017). Improving Land Use/Cover Classification with a Multiple Classifier System Using AdaBoost Integration Technique. Remote Sens..

